# A case study in connectomics: the history, mapping, and connectivity of the claustrum

**DOI:** 10.3389/fninf.2014.00083

**Published:** 2014-11-11

**Authors:** Carinna M. Torgerson, John D. Van Horn

**Affiliations:** Department of Neurology, Laboratory of Neuro Imaging, Institute of Neuroimaging and Informatics, University of Southern CaliforniaLos Angeles, CA, USA

**Keywords:** claustrum, connectomics, macro-scale, micro-scale, Wilson's Disease, consciousness

## Abstract

The claustrum seems to have been waiting for the science of connectomics. Due to its tiny size, the structure has remained remarkably difficult to study until modern technological and mathematical advancements like graph theory, connectomics, diffusion tensor imaging, HARDI, and excitotoxic lesioning. That does not mean, however, that early methods allowed researchers to assess micro-connectomics. In fact, the claustrum is such an enigma that the only things known for certain about it are its histology, and that it is extraordinarily well connected. In this literature review, we provide background details on the claustrum and the history of its study in the human and in other animal species. By providing an explanation of the neuroimaging and histology methods have been undertaken to study the claustrum thus far—and the conclusions these studies have drawn—we illustrate this example of how the shift from micro-connectomics to macro-connectomics advances the field of neuroscience and improves our capacity to understand the brain.

## Introduction

Macro-scale connectomics, the study of neuronal connections between two or more regions of the brain, combines the principles of functional specialization and functional integration. While there are only a mere 30,000–40,000 protein-encoding human genes, and nearly 1.5 million single nucleotide polymorphisms (SNPs), there may be an astonishing 10^15^ neuronal connections in the human brain (Lander et al., 2001; Sporns et al., 2005). Despite such complexity, the use of modern neuroimaging is ushering in a new vision for describing the wiring of the brain. While graph theory, diffusion imaging, and even functional imaging are still in their relative infancy, researchers have never possessed more appropriate tools for decoding the enigmas of the brain. Connectomics analysis is particularly revelatory for small, highly connected structures, like the claustrum.

Indeed, the claustrum serves as an informative case study for examining the boon that connectomics research has brought to the field of neuroscience; it has remained one of the most mysterious structures in the brain since the 17th century. Its name, in fact, means “hidden away” (Crick and Koch, 2005). Now, macro-scale connectomics allow for new analysis that may help unlock the secrets of the enigmatic structure. In this literature review, we discuss the limitations that have led to the claustrum to be investigated through a connectomics lens in the human and in other animal species, even before Sporns, Tononi, Hagmann, Bullmore, and others launched the modern connectomics movement. Our review includes descriptions of neuroimaging and histology studies tracing claustral connectivity, its putative role(s) in various neural systems, its reported influence in neurological syndromes, and examines the recent flurry of interest in the macro-connectomics of the claustrum.

## Background

**Figure 1:**
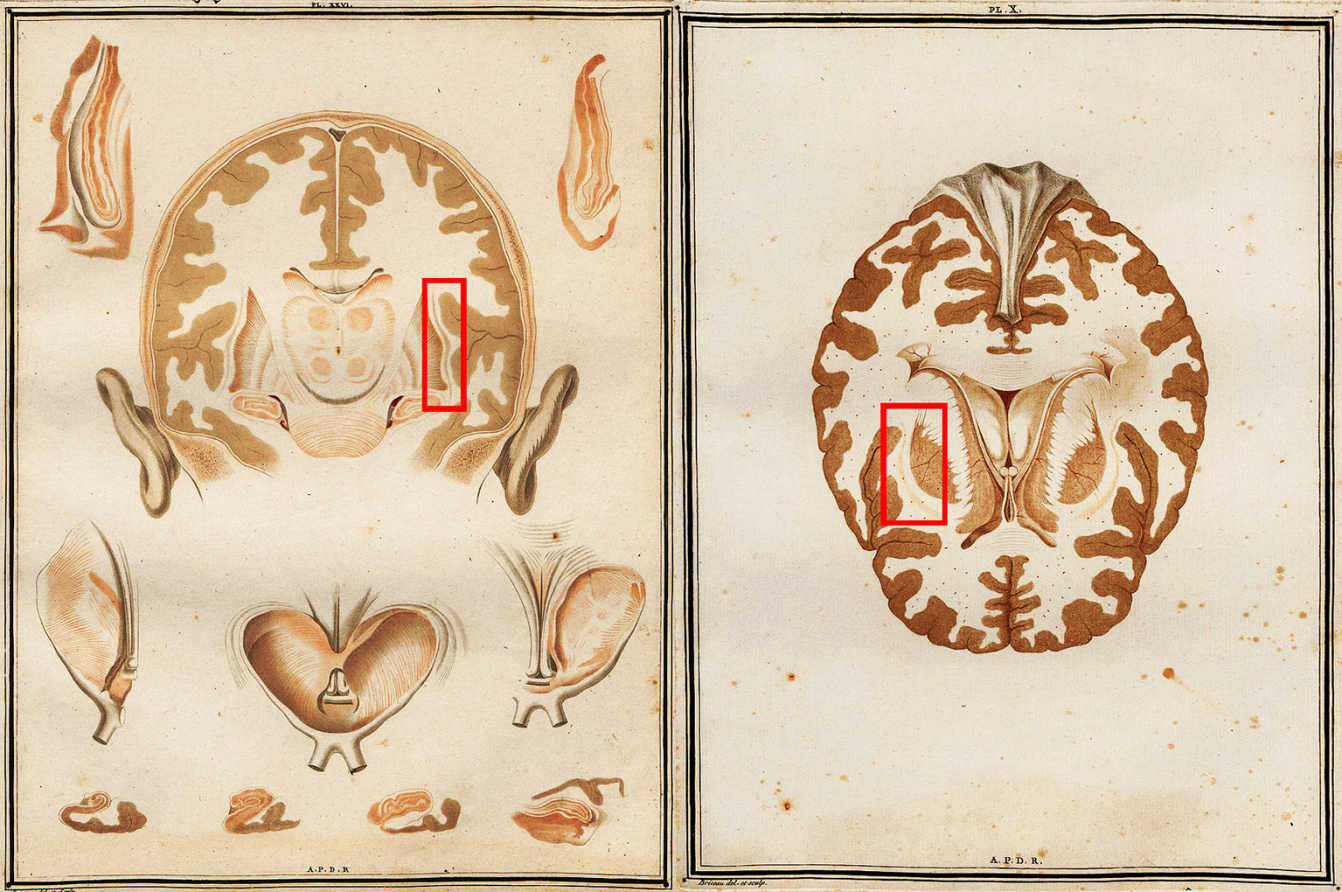
**Two early views of the brain which feature the human claustrum from Vicq D'Azyr's *Traité d'anatomie et de physiologie***. Red boxes applied by the authors to indicate the location of the left and right claustra.

The structure of the claustrum is visible as early as 1672 in the drawings of Thomas Willis (Bayer and Altman, 1991) who first proposed that higher cognitive functions arose from the convolutions of the cerebral cortex, rather than the ventricles (Molnár, 2004). Karl Friedrich Burdach, first described the claustrum (using the German word “vormauer”), however, in his seminal work *Von Baue und Leben des Gehirns*, in the early 19th century (Parent, 2012). Burdach himself credited the first illustrated depiction (Figure 1) of the structure to the 1786 drawings by Marie-Antoinette's personal physician, Félix Vicq-d'Azyr, who not only discovered the *substantia nigra*, but also provided the most detailed drawings of the basal ganglia of his time (Parent, 2012). Perhaps the first person to appreciate how crucial the claustrum is in multi-modal processing was Theodor Meynert, the director of the psychiatric clinic at the University of Vienna in the late 19th century. Investigating aphasia, Meynert noted that many post-mortem examinations of aphasic patients turned up pathological changes between the insula and the Sylvian fissure. The general belief at the time held that the entire cortex surrounding the Sylvian fissure was dedicated to speech. Meynert hypothesized that the claustrum contained an “acoustic field” that corresponded to the beginning of the *Acousticusstrang*, or “acoustic tract” (Eling, 1994). Information from the acoustic nerve, he posited, was associated with the speech system through spindle-shaped association cells in the claustrum, before being transmitted to the Sylvian fissure. His evidence for this relationship was the well-understood relationship between the claustrum and other “association systems” in the brain (Eling, 1994).

Classification of the claustrum is not a straightforward process. It cannot be accurately described as strictly cortical or subcortical, as it possesses the laminar organization and characteristic pyramidal somata of cortical regions in Insectivora (Narkiewicz and Mamos, 1990), but also contains some notably subcortical cell types (Mathur et al., 2009). Although prominent researchers such as Brodmann and Wernicke suggested that the claustrum represents the innermost layer of the insula due to its close proximity (Landau, 1919) it is somewhat unsatisfactorily included as part of the basal ganglia today. As a case in point, a search for the claustrum on PubMed will automatically also search for the broader term “basal ganglia,” presumed to reflect the fact that claustral afferent inputs are believed to be similar to the striatum, although its efferents connect directly to the cortex without passing through a thalamic relay (Salerno et al., 1984). These efferent connections have a relatively slow conduction speed, which suggests they are small in diameter and/or poorly myelinated. More recently, Pirone et al. (Pirone et al., 2012) have concluded that the more likely ontological relationship of the human claustrum is with insula, rather than basal ganglia, based on immunostaining of human tissue. The detailed overview provided by Druga (2014) indicates a pallial origin of the claustrum, whereas the striatum appears to be of sub-pallial origin. Puelles (2014) notes that various techniques throughout history have suggested the claustrum could be derived from insular cortical strata, the subpalium/basal ganglia, non-insular pallium, or even that different claustral sub-divisions arose from distinct regions. He does, however, note that recent genoarchitectonic and immunocytochemical investigations seem to have reinstated the insular derivative theory. Not only do these new investigations concur with the claustral differences noted in species with and without extreme capsules, they also bolster the recent discussions of the role of the claustrum in consciousness (see Methods for Studying the Claustrum, below), since the insula itself has been recently implicated as a neural site for conscious awareness (Craig, 2009).

**Figure 2:**
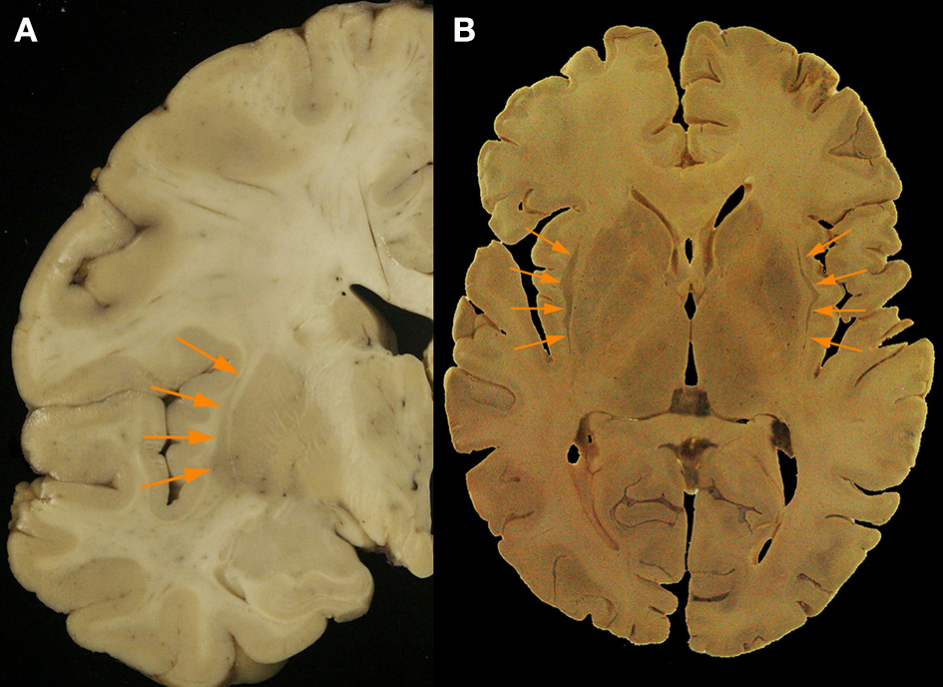
**(A)** Axial post mortem anatomical view of the claustrum (orange arrows; taken from the Rasmussen Neuroanatomical Collection housed at the UCLA David Geffen School of Medicine, Los Angeles, CA), and **(B)** a separate coronal view of the claustral body in the left hemisphere from post-mortem tissue (orange arrows). Modern neuroimaging computational segmentation algorithms do not consider the claustrum due to the particular difficulty of extracting it from the surrounding tissues.

Despite its considerable—and controversial—history, the structure itself is diminutive (Figure 2A). As represented in the Talairach and Tournoux (1988) atlas, the claustrum is located medial to the insular cortex and lateral to the putamen from between −4 and +16 mm relative to the AC-PC plane. The right claustrum has an approximate average surface area of 1551.15 mm^2^ and volume of 828.83 mm^3^ while the left claustrum has a surface area of 1439.16 mm^2^ and volume of 705.82 mm^3^ (Kapakin, 2011). The noticeable asymmetry (Figure 2B) in structure, volume, and average anisotropy (Cao et al., 2003) may relate to function, as the right claustrum, but not the left, has been shown to react differently to congruent and incongruent stimuli (Naghavi et al., 2007). Although the fine-grained anatomy of the claustrum in the human remains poorly understood, it is reasonable to expect that there exist major divisions present as in all of the species examined to date, i.e., a ventral, “endopiriform” and a dorsal “insular” component. Given a putative shared history with the insula, neural sub-divisions may be present mirroring, in part, those of adjacent insular cortex (Puelles, 2014). Thus, the claustrum is unlikely to function as a single uniform body, *per se*, but have finely interconnected sub-divisions. Lastly, it remains unclear whether blood arrives through the vessels penetrating the insula (Edelstein and Denaro, 2004) or from the deep and superficial sections of the middle cerebral artery (Crick and Koch, 2005).

Embryonic observation shows that neurons settle in the claustrum 4–6 days after the peak of neurogenesis (Bayer and Altman, 1991). As most structures do with age, the claustrum increases in volume through middle age and decreases again in old age (Wisco et al., 2008). It shares its ontogeny with the insular cortex, but not with another of its neighbors, the putamen (Pirone et al., 2012). The claustrum is very closely related to both the insula and external capsule. In fact, removal of the white fibers in the external capsule leads to the dorsal claustrum is also being removed. Removing all the fibers from the external capsule that merge in the dorsal claustrum leads to removal of the dorsal claustrum, leaving the putamen exposed, without a lateral covering (Fernandez-Miranda et al., 2008b). The dorsal external capsule is mainly composed of projection (intra-hemispheric cortico-subcortical) and not association (intra-hemispheric inter-lobar cortico-cortical) fibers (Fernandez-Miranda et al., 2008a).

## Methods for studying the claustrum

The claustrum has been the subject of a relatively small body of research, although the pace of research is increasing in today's era of diffusion imaging and macro-connectomics (Table 1). In fact, many studies that mention the claustrum only find its involvement to be a result of their research question, but do not intend to focus on the structure, nor define it particularly carefully. Until recently, the literature was predominantly composed of animal studies (Table 2). Human *in vivo* claustrum studies seem to be a phenomenon almost exclusive to the 21st century. While the paucity of neuroimaging technology throughout most of history can account in some part for the scarceness of claustral studies, high-resolution imaging of other small, non-cortical structures was undertaken far earlier. The majority of claustrum investigations have examined the microscopic detail of the claustrum: its neuronal composition (Sherk and LeVay, 1981; Bayer and Altman, 1991; Mathur et al., 2009; Smythies et al., 2012b); its afferents to a single cortical region (Edelstein and Denaro, 2004; Smith et al., 2012); and its excitatory or inhibitory properties (Sherk and LeVay, 1981; Shima et al., 1996). Only a relative few at the time of this writing have attempted to create a macro-scale picture of how the claustrum fits within a larger context—some more recent studies attempt to discover its role in cortico-cortical networks (Hadjikhani and Roland, 1998; Tanne-Gariepy et al., 2002; Poeppl et al., 2014) or to understand its function by analyzing how it alters the electrical frequency of incoming signals (Smythies et al., 2014).

**Table 1:** **Approaches to studying the claustrum by year and by method**.

**Author(s)**	**Year**	**Method**	**Regions**	**Animal**	**# of subjects (human only)**	**Journal**
Landau (Landau, 1919)	1919	Sectioning, review	Nucleus amygdalae, substantia perforata anterior, olfactory cortex	Human	Unclear	Journal of Anatomy
Berke	1960	Cortical ablation, electrical coagulation	Temporal area 22, premotor cortex, rostral frontal cortex—considers most fibers to be “fibers of passage”	Macaque monkey		Journal of Comparative Neurology
Spector (Spector et al., 1970)	1970	EEG, EMG, EOG	Medial lemniscus, primary auditory cortex, somatic sensory area II, lateralis posterior, centrum medianum, ventralis lateralis	Cat		Experimental Neurology
Carey (Carey et al., 1979)	1979	Horseradish peroxidase	Visual cortex, frontal eye fields	Tree shrew and Senegal bush baby		Journal of Comparative Neurology
Carey (Carey et al., 1980)	1980	Horseradish peroxidase	Striate cortex: layer IV, layer IIIb, layer VI, layer I, areas 17, 18, and 19	Tree shrew		Brain Research
LeVay and Sherk (LeVay and Sherk, 1981a,b)	1981	Golgi preparations, electron microscopy, anterograde, and retrograde tracers	Lateral hypothalamus, nucleus centralis thalami, medial geniculate nucleus, lateral posterior-pulvinar complex, midbrain, locus coeruleus, dorsomedial thalamic nucleus, substantia nigra, mesencephalic reticular formation, optic radiation, splenium of the corpus callosum, area 17, area 18, area 19, posteromedial lateral suprasylvian area, posterolateral lateral suprasylvian area, area 20a, area 20b, area 21a, dorsal lateral suprasylvian area, ventral lateral suprasylvian area, lateral geniculate nucleus	Cat		The Journal of Neuroscience
Pearson (Pearson et al., 1982)	1982	Horseradish peroxidase	“Entire cortex,” precentral gyrus, need advice: all are listed as area “#”	Monkey		Brain Research
Markowitsch (Markowitsch et al., 1984)	1984	Horseradish peroxidase and autoradiography				
Arikuni and Kubota (Arikuni and Kubota, 1985)	1985	Horseradish peroxidase	Caudate nucleus, pulvinar complex, amygdaloid complex	Macaque monkey		Neuroscience Research
Carey and Neal (Carey and Neal, 1985)	1985	Anterograde and retrograde tracers	Area 18b, not area 17	Rat		Brain Research
Carey and Neal (Carey and Neal, 1986)	1986	Anterograde and retrograde tracers	Thalamus, hypothalamus, intralaminar nuclei, area 17	Tree shrew		Brain Research
Witter (Witter et al., 1988)	1988	Anterograde and retrograde tracers	Paralimbic and limbic cortical areas, cingulate cortex, preirhinal cortex, insular cortex, subicular complex, entorhinal cortex, olfactory areas, orbitofrontal cortex, prepiriform cortex	Cat		Neuroscience
Bayer and Altman (Bayer and Altman, 1991)	1991	Long-survival [^3^H]thymidine	Limbic cortex, cortical layer VIa, cingulate cortex, visual cortex, motor cortex, medial prefrontal cortex, perirhinal/insular cortex	Rat		Neuroscience
Minciacchi (Minciacchi et al., 1995)	1995	Retrograde fluorescent tracers	V1, S1	Cat		Journal of Comparative Neurology
Kowianski (Kowianski et al., 1999)	1999	Cresyl violet staining	Entorhinal cortex, hippocampus, limbic system, note: not a connectivity study but discusses the implications of results in terms of other connectivity studies	Sorex, rat, mouse, guinea pig, rabbit, cat, macaque, cercopithecus, human	5 humans	Brain, Behavior, and Evolution
Kowianski (Kowianski et al., 2000a)	2000	FluoroGold labeling (retrograde tracer)	Motor cortex, somatosensory cortex, auditory cortex, visual cortex	Rabbit		Annals of Anatomy
Mohapel (Mohapel et al., 2000)	2000	Lesioning	Amygdala	Rat		Epilepsia
Tanne-Gariepy (Tanne-Gariepy et al., 2002)	2002	Retrograde tracers	M1, pre-SMA, SMA-proper, PM, and area 46	Macaque monkey		The Journal of Comparative Neurology
Edelstein (Edelstein and Denaro, 2004)	2004	Review	Nucleus medialis dorsalis, reticularis thalami, dorsal occipital cortex, temporal poles, area 17, area 18, area 19, parahippocampal gyrus, Clare-Bishop area, putamen, zona incerta, dorsomedial thalamus, suprageniculate thalamus, proreate gyrus, frontal eye fields, middle suprasylvian gyrus, anterior lateral gyrus, hippocampus, subiculum, nuclei pontis oralis, pontine parabrachial nuclei, cingulate cortex	Various		Cellular and Molecular Biology
Chachich (Chachich and Powell, 2004)	2004	FluoroGold labeling, electrophysiological single-unit recordings, lesioning	Thalamus, mPFC, entorhinal cortex, subicular cortex, amygdala, caudate putamen, insular cortex, parietal cortex	Rabbit		Behavioral Neuroscience
Crick and Koch (Crick and Koch, 2005)	2005	Review	Motor cortex, prefrontal cortex, cingulate cortex, visual cortex, temporal and temporopolar cortices, parietooccipital and posterior parietal cortex,	Various		Philosophical Transactions of the
			frontoparietal operculum, somatosensory areas, prepiriform olfactory cortex, entorhinal cortex, hippocampus, amygdala, caudate nucleus			Royal Society B: Biological Sciences
Fernandez-Miranda (Fernandez-Miranda et al., 2008b)	2008	Klingler fiber dissection and DTI	External capsule, amygdala, prepiriform cortex	Human	15	Journal of Neurosurgery
Fernandez-Miranda (Fernandez-Miranda et al., 2012)	2012	High-definition fiber tracking (HDFT)	External capsule does not connect, but in fact forms a false continuation loop	Human	6 healthy controls, 36 patients (HDFT); 20 healthy controls (fiber dissection)	Neurosurgery
Park (Park et al., 2012)	2012	HARDI	Olfactory bulb, entorhinal cortex, putamen, globus pallidus, olfactory tubercle, prefrontal cortex, premortor cortex, parietal cortex; functional association with the frontal cortex, cingulate cortex, supplementary motor area, parietal cortex, and visual cortex	Lemur		Frontiers in Neuroanatomy
Grasby (Grasby and Talk, 2013)	2013	Excitotoxic lesioning	Frontostriatal circuits	Rat		Brain Research
Milardi (Milardi et al., 2013)	2013	CSD tractography	Prefrontal cortex, visual areas, sensory-motor areas, auditory cortex, caudate nucleus, putamen, globus pallidus, corpus callosum	Human	10	Cerebral Cortex
Fauvel (Fauvel et al., 2014)	2014	MRI and rsfMRI	Right inferior frontal gyrus	Human	33	NeuroImage
Patzke (Patzke et al., 2014)	2014	Anterograde and retrograde tracers	Visual areas 17, 18, 29, and 21, temporal visual areas 20a, 20b, and AEV, contralateral claustrum	Ferret		Frontiers in Systems Neuroscience

**Table 2:** **Claustrum studies in non-human species**.

**Animals**	**Authors**	**Identifying structural observations**	**Subcortical connectivity observations**	**Major cortical connectivity observations**	**Cortical layer connectivity observations**
Carnivores	Buchanan (Buchanan and Johnson, 2011)	Fat, hook-like anterior wraps around small anterior insula. Nearly vertical in posterior. Large, triangular claustral expansion spanning the sulcal fundus leads to thin cell bridge with claustral root. Often stretches beyond insula	Insular cortex meets its superior operculum in the superior claustrum		
Cat	Kowianski (Kowianski et al., 1999)	Dorsal part is triangular and narrows ventrally, surrounding anterior rhinal fissure. Posteriorly, lies more vertically			
	LeVay (LeVay and Sherk, 1981a,b; LeVay, 1986)		Nucleus centralis thalami and the lateral hypothalamus are afferents	Primary auditory cortex appears not to project to claustrum. Roughly 290,000 cells might project to the visual claustrum	
	Minciacchi (Minciacchi et al., 1985)			Claustral projections are most prominent extrathalamic pathway to S1 and V1	
	Salerno (Salerno et al., 1981, 1984, 1989)		“Massive projections” from the claustrum to the putamen are characteristic of cats	Directly connected to cerebral cortex. Does not rely on relay efferents	
	Tsumoto (Tsumoto and Suda, 1982)			Direct projection from the dorsocaudal claustrum to the striate cortex	
Dolphin	Buchanan (Buchanan and Johnson, 2011)	Very thin. Follows characteristically extended insula of dolphins to fill insular gyri with claustral islets. Terminates far before the posterior extent of the insula			
Ferret	Patzke (Patzke et al., 2014)		Connectivity to the LGN	Areas 18, 19, 20a, 20b, and 21, the posterior parietal rostral and caudal visual areas. Connections to the contralateral claustrum were seen in the posterior parietal rostral visual area and the anterior ectosylvian visual area	
Hyrax	Buchanan (Buchanan and Johnson, 2011)	Large, broad claustrum with thin cell bridge to endopiriform root			
Lemur	Park (Park et al., 2012)		Putamen, globus pallidus, olfactory bulb, olfactory tubercle		
Llama	Buchanan (Buchanan and Johnson, 2011)	Begins anterior to insula. Claustral cells accumulate in lateral gyri, between U-fibers, and underlying fasciculi of the extreme capsule. Terminates in the superior insular operculum			
Manatee	Buchanan (Buchanan and Johnson, 2011)	Ill-defined, “wispy” claustrum. No endopiriform root			
Marsupials	Buchanan (Buchanan and Johnson, 2011)	Farther anterior of putamen than other species. A ball-like collection of cells in continuity with the endopiriform cells, flattening into a laminar shape			
Monkey	Pearson (Pearson et al., 1982)			Projections to S1 and 4, to 5 and 6, and to 7 and 9, all respectively overlap antero-posteriorly. Dorso-ventral overlap between projections from parietal lobe to S1 with 5 and 5 with 7, and between frontal lobe connecting 4 with 6 and 6 with 9	
Monkey (rhesus)	Berke (Berke, 1960)		Contended connections between the claustrum and the external capsule, and putamen. Fibers from the inferior thalamic peduncle turn into the basal claustrum	Contended connections between the claustrum and the corpus callosum and anterior commissure	
	Mufson (Mufson and Mesulam, 1982)			Receives cortical afferent input from same set of areas that project into the insula	
	Arikuni (Arikuni and Kubota, 1985, 1986)	Rostrally buried deeply in the white matter of the orbito-frontal cortex and lies caudally between the insular cortex and the putamen	Projects ipsilaterally to the pulvinar complex and has reciprocal connections with the ipsilateral amygdaloid complex	Network between the prefrontal cortex, the claustrum, and the caudate nucleus. Neuronal circuit consists of these three connections: prefronto-caudate, prefrontoclaustral, and claustro-caudate projections	
	Tanné-Gariépy (Tanne-Gariepy et al., 2002)			Projections to M1, pre-SMA, SMA proper, and various subdivisions of PM and area 46 generally originate from rostrocaudal extent. Each claustral neuron projects to only one cortical area	
Monotremes	Ashwell (Ashwell et al., 2004)	Small rounded structure embedded in the anterior commissure may constitute the claustrum in the echidna			
	Buchanan (Buchanan and Johnson, 2011)	No claustrum, except echidna, which consists merely of small root adjacent to the endopiriform group			
	Butler (Butler et al., 2002)	No claustrum or separate laminar structure can be identified			
Mouse/Rat	Kowianski (Kowianski et al., 1999)	Dorsal and ventral parts of the nucleus are separated at the level of the rhinal fissure			
Owl Monkey	Buchanan (Buchanan and Johnson, 2011)	Superior insula is claustrum-less throughout its extent			
Pig	Buchanan (Buchanan and Johnson, 2011)	Expands much further along cortex than any other species. Anterior tail reaches superior operculum. Posterior widens dramatically and appears striated. Posterior consists of triangular accumulation of cells in the superior operculum and the posterior tail reaches the ectosylvian gyrus			
Rabbit	Buchanan (Buchanan and Johnson, 2011)	Club-shaped and similar to hyrax, with a dark band of cells connecting to endopiriform root			
	Chachich (Chachich and Powell, 2004)			Reciprocal connections to neocortex and thalamus. Anterior claustrum projects to the medial prefrontal cortex	
	Kowianski (Kowianski et al., 1996, 1998, 2000a,b)	Club-shaped dorsal section that narrows inferiorly		Anteriorly connects to motor cortex, centrally dominated by somatosensory projections, posteriorly connected to auditory and visual cortices	
	Yamamoto (Yamamoto and Kawamura, 1975)		The claustrum connects to the glossopharyngeal nerve and bilateral chorda tympani		
Rat	Carey (Carey and Neal, 1985)				The major terminus is in the infra-granular layers
	Mohapel (Mohapel et al., 2000, 2001)		Connects limbic sites to the motor cortex	Compared to cat, the rat's claustrum possesses more axon collaterals that interconnect the two hemispheres	
	Sadowski (Sadowski et al., 1997a,b)			Anterior part of the insular claustrum linked mainly with the motor and prefrontal cortical areas, the central part with somatosensory fields, and posterior part with visual cortex	
	Shameem (Shameem et al., 1984)			In rat, unlike in cat, substantial proportion of cells in the dorsocaudal claustrum project to non-visual cortex	
	Sloniewski (Sloniewski and Pilgrim, 1984; Sloniewski et al., 1986b, 1987)		Recieves input from the posterior thalamus and anterior pretectum (probably throughout mammalian phylogenetic scale). Connects to zona incerta		
	Wilhite (Wilhite et al., 1986)		Connects to the diencephalic nuclei, medial septal nuclei, cingulate gyrus, subiculum, and both medial and lateral entorhinal cortex	Reciprocal monosynaptic connections exist between the claustrum and the entorhinal cortex and influence activity in the hippocampus	
Squirrel Monkey	Buchanan (Buchanan and Johnson, 2011)	The posterior superior insula is claustrum-less. Anteriorly invades the frontal operculum			
Tree shrew	Carey (Carey and Neal, 1986)		Lateral intermediate nucleus of the visual thalamus is reciprocally connected with the claustrum		The major terminus is in the granular layer and in cortical layer IV, and to a lesser extent in layers IIIb, VI, and I
	Carey (Carey et al., 1979)		P*arallel visual pathways* travel through the LGN, pretectal nuclei, claustrum, and intralaminar nuclei		
Xenarthrans	Buchanan (Buchanan and Johnson, 2011)	Anterior ovoid mass flattens posteriorly between expanding putamen and the superficial cortex. Has an endopirifom root			
Zebra	Buchanan (Buchanan and Johnson, 2011)	Does not wrap around rhinal sulcus anteriorly. Anteior accumulates thickly in spaces between the U-fibers connecting the local gyri. Posteriorly, superior portion thickens into triangular mass in the gyral cores			

Some unique characteristics have held back research of the claustrum. It is important to note that imaging of the structure is infamously challenging. Early neuroscientists often examined the brains of the deceased in order to relate abnormalities with some functional deficit the patient experienced during life. The claustrum cannot be studied in this way, as researchers have yet to induce a lesion affecting the claustrum without also affecting neighboring structures, although some natural lesions have been reported in epileptic subjects (Sperner et al., 1996; Duffau et al., 2007). Even with the advent of histological staining techniques, it is incredibly difficult to localize an injection in the claustrum without some tracer spreading. The emergence of neuroimaging could not address this limitation of size, as fine-scale structures such as the tiny claustrum can be severely distorted by high-resolution MRI (Konukoglu et al., 2013). Conversely, the claustrum is simply not visible in some low-resolution MR imaging; Meng et al. note that in the developing brain the claustrum is not visible even at 11.7-T MRI, but can be seen on T2-weighted images at 7T (Meng et al., 2012). The difficulty of capturing the claustrum has in fact helped researchers compute signal-to-noise and contrast-to-noise ratios for image restoration because it can only be seen under specific conditions (Konukoglu et al., 2013). In terms of functional imaging, temporal resolution is already notoriously poor. It is not unusual for fMRI spatial resolution to be larger than the width of the claustrum, and therefore attributing any function to just the claustrum runs the risk of ascribing tasks actually carried out in the insula or external capsule to their claustral neighbor. Such misattribution is particularly dangerous in light of the suggestions that the claustrum and insula connect to very different regions (Park et al., 2012).

**Figure 3:**
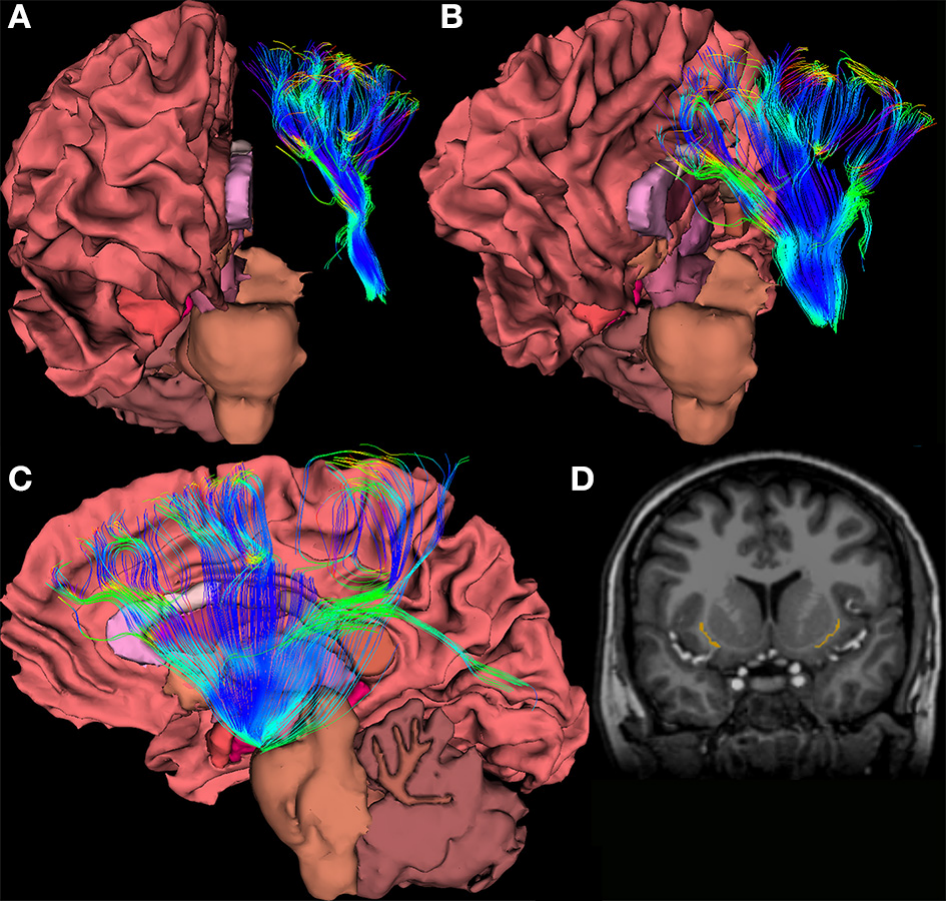
**(A–C)** Views of a 64-direction diffusion tensor (DTI) fiber tract reconstruction from a 3.0 Telsa Siemens MRI Trio scanner in an example subject showing white matter pathways emanating from a the region of the human claustrum. **(D)** The image shows a binary label mask drawn of the left and right claustra on a T1-weighted image of the same subject.

Within the last 5 years, tractography studies of the claustrum have been undertaken in hopes of obtaining a broader picture of how the claustrum relates to the rest of the brain. An example rendering based upon diffusion imaging of the claustrum is shown in Figures 3A–D. Researchers have begun to examine how the claustrum connects functionally disparate cortical networks, and to attempt to extrapolate from these individual examples a larger idea of the role the claustrum plays in the brain. Presumably, the logic of this is that the claustrum performs the same function for all networks in which it participates. The strategy for assessing function seems to be to analyze networks and structures that we understand well, and attribute any unexplainable interaction to the claustrum. This process creates a reactionary chain of research in which one aspect of claustral function is asserted, and then argued against with evidence from a different claustro-cortical network. For example, the reciprocal connections from the visual cortex to the claustrum have been used to suggest segregation of function in the claustrum, and yet, analyses of multiple sensory networks has led researchers to conclude that the claustrum functions primarily as a relay station between major networks (Minciacchi et al., 1995).

After centuries of analyzing individual connections, connectomics offers us the opportunity to analyze all connections to and from the claustrum and determine the weight of influence these have on the overall function of the structure. Such a gestalt view could shed light on the role of the claustrum, perhaps even providing a more precise definition of consciousness itself. Some researchers have eschewed a formal definition of consciousness, lest it result in biasing primary research or its results (Crick and Koch, 1990), while others list necessary components—such as an awareness of one's own physical and sentient existence (Craig, 2009), or varying awareness to unchanging stimuli (Blake et al., 2014)—but researchers seem to agree that conscious processes have access to a multitude of sensory information and some processing capacity. Characterization of a network or region in possession of these properties would almost certainly benefit from some graph theoretical calculation of the relative weights of network influence.

Still, reliance on newer investigative techniques will require an accumulation of many large studies in order to find certitude in our conclusions. In terms of DTI investigations, further knowledge of the anatomy of multi-orientational fiber populations may be necessary to increase accuracy; when axons are not oriented in a coherent fashion, so that then the voxel-averaged estimate of orientation cannot accurately summarize the orientation of the underlying fibers, continuity may be assumed between the fibers where there is none (Fernandez-Miranda et al., 2008a). Modern, *in vivo*, human imaging studies of the claustrum still suffer from low statistical power due to small sample sizes. Number of diffusion directions, voxel size, magnetic field strength, and eddy current correction will all need to be carefully controlled to obtain accurate results in a structure so small. In the long-term, parcellation algorithms (like FreeSurfer reconstruction; http://surfer.nmr.mgh.harvard.edu/) will need to become more precise if we hope to perform population-level analyses of small structures.

## Structural micro-connectomics

“The central rationale for human connectomics builds on the premise that structural brain connectivity can serve as a basis for understanding brain dynamics and behavior” (Behrens and Sporns, 2012). Despite never having been subjected to network theory analysis, many observations of connections between the claustrum and individual regions or networks have helped establish some facts about claustral micro-connectomics. Hagmann et al. (2008) have applied community detection or modularity analysis to demonstrate—using diffusion imaging and resting state functional MRI—that a close relationship exists between structural connections and functional connections. Therefore, an analysis of the structural connections of the claustrum ought to allow us to hypothesize the functional role it may play in the networks to which it contributes. Functional imaging studies will be discussed in the following section.

Inputs to the claustrum arrive from a plethora of brain areas before being assimilated, integrated, and signals directed to the claustrum (Edelstein and Denaro, 2004). These functions allow rapid adaptation to nuanced changes in one's environment. Intra-claustral interactions, which may involve dendro-dendritic synapses and networks of gap junction-linked neurons, are thought to be abundant (Smythies et al., 2012a,b), though the presence of connexin proteins would be a prerequisite for such networks which requires further exploration. Several authors have also found connections to the corpus callosum and anterior commissure (Berke, 1960; Milardi et al., 2013), although there are only one fifth as many contralateral projections as ipsilateral ones (Markowitsch et al., 1984). There is substantial evidence for claustrum-subcortical connections in the existing animal literature (for instance, in the cat, rat, and a range of insectivora, respectively; Kaufman and Rosenquist, 1985; Sloniewski et al., 1986a; Narkiewicz and Mamos, 1990; Dinopoulos et al., 1992), though there is a paucity of similar findings from *ex vivo* studies of humans. There is substantial evidence for claustrum-subcortical connections in the existing animal literature, though there is a paucity of similar findings from *ex vivo* studies of humans.

When multiple inputs converge onto the claustrum, this results in a new signal, which demonstrates integration (Edelstein and Denaro, 2004). It is particularly useful in cross-modal matching; for example, it is active when a subject sees and touches something, but not active when two items are seen or two items are touched (Arnow et al., 2002). Information can also be redirected throughout the brain by this structure (Edelstein and Denaro, 2004). In a study using macro-electrodes, the organization of somatic sensory, auditory, and visual projections to the claustrum were found to display heterotopic and multi-sensory convergence characteristics (Spector et al., 1970). Responses differ when the stimulus site or receiver site change, which indicates it is a non-homogeneous multisensory structure with three electro-physiologically distinct parts (Spector et al., 1970). Claustral neurons appear to discriminate and associate between intermodal and intra-modal sensory stimuli (Spector et al., 1974). The neurons of the striate cortex and pyramidal tract show decreased spontaneous firing during stimulation of the claustrum, which indicates it may play a role in regulating afferent sensory information (Edelstein and Denaro, 2004). Claustral cells themselves display very low spontaneous discharge (Spector et al., 1974). Connections to limbic regions seem to indicate that the claustrum possesses other functions beyond sensory integration. The amount of intra-modal branched inputs is higher in the claustrum than in relay nuclei, which indicates that the claustrum must do more than simply relay information (Minciacchi et al., 1995). Zingg et al. (2014) suggest that the claustrum may provide additional means of direct interaction between neural sub-networks. The structure and function of claustral connections may differ to some extent between genders. For example, the claustrum it is active during penile stimulation, but not clitoral stimulation (Georgiadis et al., 2009), and the level of activation corresponds to turgidity of the penis (Arnow et al., 2002). In fact, the claustrum and the brain stem are the only two brain regions active in males during sexual arousal, but inactive during competitive arousal (Redoute et al., 2000).

While the claustrum as a whole is known for multimodal processing, most neurons within it are not multisensory processors (Remedios et al., 2010). Because of this, it has been implied that perhaps claustrum synchronizes cortical regions that are responsible for bilaterally coordinated behaviors such as eye movements without actually changing the data being shuttled through itself (Smith and Alloway, 2010). Another hypothesis is that the claustrum seems to work to counterbalance the homunculus of the brain; overrepresented regions in S1 and V1 are relatively underrepresented in the claustrum and a preference exists for retinal periphery representation (LeVay and Sherk, 1981b; Minciacchi et al., 1995). Sherk and LeVay suggest that claustral efferents serve as inhibitors in order to shape the receptive field properties of cortical neurons (Sherk and LeVay, 1981; Shima et al., 1996). Furthermore, the claustrum may play a crucial role in plasticity and reorganization. Such a function would require the claustrum to recognize the unknown modular specific code carried by an afferent axon, or require claustral efferent to return to the same neuron (or neuronal group) that gave rise to the afferent axon (Smythies et al., 2012b).

Micro-connectomics of the claustrum was, until fairly recently, largely studied through retrograde tract-tracing, since the proximity of the claustrum to other areas makes placement of strictly claustral anatomical tracers incredibly imprecise. Unfortunately, retrograde tract-tracing requires clearly defined anatomical boundaries, which are still under investigation in the claustrum (Mathur et al., 2009).

## Macro-connectomics

Sexuality studies may offer the easiest starting point for understanding the interactions between claustrocortical regions. Firstly, the claustrum is activated during penile, but not clitoral arousal (Georgiadis et al., 2009). Men and women tend to experience sexual arousal differently in response to visual stimuli, with one of the most notable differences being that visual stimulation is associated more with male arousal (Redoute et al., 2000; Hamann et al., 2004)—though this assumption has recently been questioned by Rupp and Wallen (2008). Arnow et al. (2002) suggest the claustrum may facilitate reflexive cross-modal transfer of visual input to imagined tactile (penile) stimulation. In both PET and fMRI investigations, claustrum activation corresponded to penile turgidity with statistical significance (Arnow et al., 2002; Stoleru et al., 2012). There is some question whether such activation is specific to sexual arousal, or may be indicative of more broad motivational processing; some imaging has shown claustral activation correlated with thirst, hunger, and emotional motivation (Stoleru et al., 2012).

A further breakdown of this relationship has been undertaken to separate sub-networks in which the claustrum participates during co-activation of the networks. It was demonstrated psychosexual arousal was characterized by lateral prefrontal cortex, superior parietal lobule, and medial and inferior frontal gyri (all bilaterally), while physiosexual arousal was characterized by anterior cingulate cortex (which the authors consider to be alternatively called ventromedial prefrontal cortex and medial orbitofrontal cortex) (Poeppl et al., 2014). The one region that was activated during both types of arousal was the claustrum. Furthermore, the right claustrum/insula was the only field shown to be involved in visual-tactile stimulation, but not activated in either pure tactile or pure visual stimulation (Hadjikhani and Roland, 1998). Due to observational analysis, researchers have long suspected that sexual stimuli and autonomic processes must be linked in the brain in a way that allows for two-way communication, and meta-analytic review suggests this link may be the claustrum and the putamen. Given its multimodal, cortical and cross-cortical connections, the claustrum seems like a viable candidate for cross-modal matching, such that modality-specific areas can communicate with the claustrum via unimodal connections, which can then be exchanged and altered to output a novel unimodal signal (Smythies et al., 2014). Additional functional imaging investigations of sexual arousal at ultra-high field and spatial resolution would provide confirmation of the psychophysiological role of the claustrum in concert with other brain regions believed to be different between males and females.

Claustral activation also occurs during identification of the use of a fluency heuristic, and may imply that the prioritization of certain inputs, along with the priming effect, may be driven or mediated despite its modest structural size (Volz et al., 2010). It has also been implicated in learning; the insula and claustrum are BOLD-activated during active, but not passive learning (Kersey and James, 2013). Given the prevalence of claustral abnormalities in memory disorders, the role of the claustrum in the creation of fluency heuristics may be due to its involvement in recall. If the conclusions about sensory integration are correct, an aclaustral subject could still respond to isolated stimuli, but would not be able to process complex ones or to coordinate the synchronization of inputs from multiple modalities (Crick and Koch, 2005).

In an fMRI study of musicians, Fauvel et al. (2014) found that the bilateral claustrum was functionally connected with the right inferior orbitofrontal gyrus at rest, and formed a resting-state executive control network. The authors suggested that the network integrates emotion aroused by the auditory stimuli in order to drive planning of future motor sequences that would continue to arouse the appropriate emotionalism in the music, although admitted that their functional conclusions were merely hypothetical.

Electrolytic lesions and ablations of the claustrum inhibit conditioned activity. Electrical stimulation can lead to salivation, tongue movements, blinking, and swallowing, and contralateral upper extremity motions, although results differ between stimulation of the claustral-putamen pathway and the claustral-amygdala pathway (Edelstein and Denaro, 2004). Stimulation can also increase spinal reflexes. Pupillary dilation, licking, swallowing, shivering, and ear movement have been affected by claustral lesions in cats. Lesions of the left claustrum specifically lead to eyelid twitching, myoclonic jerking, and convulsive seizures, although one must remember lesion studies in small structures are more susceptible to downstream effects and other errors (Wada and Tsuchimochi, 1997).

## Clinical syndromes associated with claustral damage and dysfunction

Table 3 lists the studies of clinical conditions where the claustrum has been implicated, along with the suspected means of claustral involvement. It is important to note that no clinical studies have been able to isolate the claustrum as the only region of involvement. This may support the idea that dysfunction in the claustrum results primarily in network disruption, rather than a specific functional deficit.

**Table 3:** **Clinical syndromes with putative claustral involvement**.

**Clinical diagnosis**	**Lead author**	**Suspected role of claustrum**
AIDS	Kozlowski (Kozlowski et al., 1997)	Regional reduction in claustral volume
Alzheimer's disease (AD)	Ogomori (Ogomori et al., 1989)	Claustral amyloid plaques present in 100% of AD patients studied, mostly type 2 plaques
Bipolar	Selvaraj (Selvaraj et al., 2012)	Reduced GM in the right claustrum
	Chen (Chen et al., 2012)	Increase in GM volume in the left claustrum
	Ford (Ford et al., 2013)	Default mode network activation in the right putamen, claustrum, and insula correlates positively with the Bipolarity Index
Dementia with Lewy Bodies (LBD)	Yamamoto (Yamamoto et al., 2007)	LB scores in the claustrum are lower than in the insular and inferior temporal cortices and amygdala, but higher than in the BA17, precentral, postcentral, and transverse temporal cortices
Extended Psychosis Phenotype	Jacobson-McEwen (Jacobson McEwen et al., 2014)	Weaker intrinsic functional connectivity (iFC) between the claustrum and dorsal anterior cingulate cortex
HIV	Smith (Smith et al., 2000)	Regional increase in normal white matter in claustrum sub-nuclear white matter
HIV-Encephalitis	Sevigny (Sevigny et al., 2005)	Astrogliosis of the claustrum and other basal ganglia
Multiple Sclerosis	Klaver (Klaver et al., 2013)	Demyelination of the claustrum found in MS patients
Parkinson's	Kalaitzakis (Kalaitzakis et al., 2009)	Claustral αSyn positivity in 75% of non-demented cases, but 100% of patients with dementia (Parkinson's w/dementia or DLB)
Schizophrenia	Shapleske (Shapleske et al., 2002)	White matter excess in the claustrum is correlated with hallucinations in schizophrenia
	Cascella (Cascella et al., 2011)	Severity of delusions is correlated with reduction in left claustral volume
	Kong (Kong et al., 2012)	GM decrease in the claustrum correlates ositively with neurlogical soft signs
Seizures	Wada (Wada and Kudo, 1997)	Lesioning of the left claustrum led to bilateral eyelid twitching, myoclonic jerking, and eventually convulsive seizures
Transitory encephalopathy	Sperner (Sperner et al., 1996)	Epilepsy and psychotic disturbance was associated with bilateral lesions of the claustrum
Wilson's disease	Sener (Sener, 1998)	Bilateral claustrum brighter and thicker in T1 (see also King et al.)
	Sener (Sener, 1993)	T2 hyperintensity in the claustrum—the “bright claustrum sign”—is a marker of the disease

This diminutive structure has been investigated for its potential role in seizure generalization (Fernandez-Miranda et al., 2008b). It is thought that epileptoform propagation from limbic sites may be linked to the motor cortex via the claustrum (Mohapel et al., 2000). A case study was conducted on a 12-year-old girl who presented with status epilepticus (multiple, repeated complex partial and myoclonic seizures occurred in the upper extremities and face with orofacial automatisms, eye deviation, and nystagmus) displaying psychotic behavior with agitation, severe cognitive impairment and temporary loss of vision, hearing and speech, as well as loss of orientation in time and place. Bilateral, strip-like lesions were discovered in T1 and T2 images of her claustrum and external capsule. The case indicates that lesions of the claustrum function more like gray matter disease than white matter disease. Additionally, the case study implies a functional correlation between seizures, behavioral state, and abnormalities in the claustrum (Sperner et al., 1996).

In contrast, unilateral removal of the claustrum did not lead to sensorimotor or cognitive impairment in patients receiving surgery for low-grade cerebral glioma (Duffau et al., 2007). So, perhaps the claustrum operates as part of a network or networks, rather than as the epicenter of a network.

The most common clinical association is the effect of the claustrum on memory. Claustral amyloid plaques accumulation has been implicated in the outcomes of Alzheimer's disease and aging (Morys et al., 1994; Fernandez-Miranda et al., 2008b). These amyloid deposits seem to cluster in the ventral claustrum, and may disrupt limbic connections (Morys et al., 1994). These changes in the claustrum associated with aging, however, are subtle, appearing several years after those in the cerebral cortex become apparent. The claustrum has also been examined in cases of memory impairment associated with HIV, AIDS, Parkinson's, and Dementia with Lewy Bodies (DLB) (Kozlowski et al., 1997; Yamamoto et al., 2007; Smith et al., 2008; Kalaitzakis et al., 2009).

Other neurological conditions have been examined in the claustrum as well. Negative correlations between anhedonia and metabolism in the claustrum have been shown in both patients with unipolar depression and bipolar disorder (BD), which may be part of overall enlargement of the basal ganglia and increase in claustral GM volume in BD (Chen et al., 2011). Such factors may be the result of differences in pruning between diseased and healthy populations. Severity of delusions in schizophrenia is correlated with the reduction in left claustral volume (Cascella et al., 2011) and schizophrenia patients with hallucinations show signs of white matter excesses (Shapleske et al., 2002). The claustrum is alternately reported as spared from insular gliomas, or commonly found to be invaded by such tumors (Fernandez-Miranda et al., 2008a).

In Wilson's disease, abnormal brightness of T2-weighted images in the claustrum is considered one of the markers of the disease though its effects may be misattributed in the literature to its close neighbor, the putamen (Sener, 1993; King et al., 1996). In fact, Wilson himself cited the possible role of the claustrum in his initial description of the disease in 1912 (though he referred to it as “Progressive Lenticular Degeneration”) (Wilson, 1912; Sener, 1993). The disease, which is invariably fatal without intervention, is caused by high copper levels—and sometimes iron levels—in the brain, particularly in the basal ganglia. Symptoms include bilateral tremors of the extremities, spasticity of the limbs and face, emaciation, dysphagia, dysarthria, emotionalism, and difficulty maintaining equilibrium (Wilson, 1912).

## Claustral involvement in neural systems

Based on the summation of the micro-connectomics information gathered from centuries of histology, structural imaging, functional imaging, and clinical pathology studies, interactions have been proposed between the claustrum and every major sensory network. The extent to which these networks rely on the function of the claustrum, or to which the claustrum relies on information from these networks, remains to be investigated.

### Visual

Claustral neurons are overwhelmingly binocular; 84% respond to stimuli from either eye, while only 40% of overall cortical neurons do (Sherk and LeVay, 1981). Responses in the claustrum to photic stimuli can be abolished by lesioning the lateral geniculate nucleus (LGN) (Edelstein and Denaro, 2004). V1 connections may not be reciprocal, as connections from V1 to the claustrum in macaques have not been reported (Crick and Koch, 2005).

Cortico-claustral connections from layer VI reach layer IV through the claustrum, creating an alternative route to the direct projection from layer VI to layer IV (LeVay and Sherk, 1981a). In the contralateral half of the visual field, the upper fields map to the caudal region of the claustrum, while the lower ones map rostrally. The far periphery can be mapped to the claustral surface, while the vertical meridian lies at the lower limit of the visual region. A single representation of the visual hemi-field exists as a unified map without discontinuities or duplications in the claustrum (LeVay and Sherk, 1981b).

### Auditory

The medial ectosylvian gyrus (AII) relays information from the medial geniculate nucleus to the claustrum. In fact, ablation of the entire AII results in a lack of claustral response to MGN stimulation (Edelstein and Denaro, 2004).

### Motor

Functional localization has not been demonstrated in the claustrum, so it is unlikely that the claustrum can influence specific muscular groups (Salerno et al., 1984).

The claustral loop to the striate cortex is involved in motion detection, but cannot discriminate the direction of motion (Edelstein and Denaro, 2004). Most (approximately 70%) of movement-related neurons increased discharge regardless of whether the motion was a push, a pull, or a turn, while only 16% were selective to one movement. This differs from the specificity of the motor cortex itself, in which about half of the neurons are responsive to a single motion (Shima et al., 1996). This may be indicative of a high degree of convergence of inputs. This lack of selectivity, along with the presence of inhibitory efferents, may suggest that the claustrum generally suppresses cortical activity immediately before the initiation of movement.

Projections to M1, pre-SMA, SMA-proper, and various subdivisions of PM and area 46 emanate from the entire rostro-caudal extent of the claustrum, with no distinct topographic or somatotopic organization. Along the dorso-ventral axis, however, these projections tend to originate in the dorsal or intermediate claustrum (Tanne-Gariepy et al., 2002). There is overlap of region of origin in most motor areas, but Pre-SMA and SMA-proper both uniquely show local segregation through inter-digitations. Area 46 also receives projections from the most ventral portion of the caudal claustrum, which sends minor projections to M1, the subareas of PM, pre-SMA, and SMA-proper (Tanne-Gariepy et al., 2002). M1 projections to the claustrum are then projected to S1, indicating a role in sensorimotor coordination (Smith et al., 2012).

In terms of oculomotor control, the mid-ventral claustrum receives projections from the frontal eye fields, and is organized topographically according to the size of saccades (Tanne-Gariepy et al., 2002).

### Somatosensory

While there is general agreement on its heterotopic organization, there is no apparent somatotopic organization of the claustrum (Spector et al., 1974). Some somatic afferents to the claustrum are conveyed via the posterior spinal funiculi fibers. In addition, the anterior ectosylvian gyrus (SII) relays information from the ventral posterolateral nucleus (VPL) to the claustrum. Lesions in the VPL lead to reduced responsivity of the claustrum to skin stimulation. Marked claustral activity was noted in response to stimuli in somatic-vagal-tooth pulp. This may be indicative of trigeminal projections similar to those noted in other structures of the basal ganglia (Edelstein and Denaro, 2004).

## Looking toward the future

Studying connectomics will improve the quality of human brain research. Firstly, connection profiles contain valuable information about a brain region. In fact, anatomical delineations between brain regions can be drawn out by examining which structural elements share similar long-range connections that differ from the connection profiles of other structures. Therefore, connectomics analysis could be a valuable tool in creating a reliable white matter atlas, a resource that does not currently exist. In fact, there is not even a universally agreed upon system of cortical parcellation (Sporns et al., 2005).

At present, the rapid proliferation of claustrum research seems to be mostly composed of macro-connectomics approaches, such as HARDI, DTI, and functional imaging. Shifting the focus, however, from micro to macro is not likely to solve all of the mysteries of the relationship between brain structure and function. Sporns and Tononi, in their noteworthy paper introducing the concept of the connectome, have argued that scientists would not be able to map structure to function in the human brain without a comprehensive connectional model (Sporns et al., 2005). A complete biophysical model of the human connectome, however, would “provide a unified, time-invariant, and readily available neuroinformatics resource that could be used in virtually all areas of experimental and theoretical neuroscience.” Several maps of the human macro-scale structural connectome have been presented, (Irimia et al., 2012a,b; Barch et al., 2013; Ugurbil et al., 2013; Van Essen et al., 2013) with additional multimodal atlases forthcoming (Amunts et al., 2014). Yet, defining which clusters of connections comprise a sub-networks and determining the hierarchy of which networks depend on each other will take a lot of additional time and analysis of both the macro- and the micro-scale.

Micro-scale approaches to assessing connectivity have several shortcomings, although most of these can be mitigated by combining micro- and macro-scale approaches. Assessments of differences in network theory measures, such as betweenness centrality and assortativity, are difficult to translate into clinically relevant knowledge (Johansen-Berg, 2013). Histological attempts to study connectomics often neglect to take into account the difference in tissue size, behavior, and integrity caused by observation and intervention. Where histology tends to underestimate axonal properties, micro-structural observations from tractography data tend to be heavily influenced by the parameters of reconstruction chosen by the researcher (Assaf et al., 2013). Therefore, the combination of macro- and micro-scale connectomics allows for validation of statistics which tend to be particularly prone to error; crossing fibers, a perennial problem for tractography analysis, can be separated according to micro-structural characteristics such as axon diameter in order to increase the validity of diffusion data. Not only does this combination improve the accuracy of existing data analytic techniques, but a combinatory approach also opens the door to new, more complex and multi-scaled analyses. Localized data can be applied to estimation of conduction velocity, which helps in assigning network weight (Johansen-Berg, 2013). Merging EEG and fMRI data with tractography and histology would allow us to take functional research conclusions out of the realm of correlation and start to understand the causation of the data patterns that have emerged in fMRI research. Uniting the two methods would undoubtedly generate further about the interplay of overlapping networks in the human brain (Assaf et al., 2013).

The claustrum appears ideally suited for synthesizing multi-modal data because it appears to be the infrastructure through which sub-networks communicate. Since its discovery, its function was assumed to be connecting disparate cortical areas. However, using modern functional imaging methods, it is highly challenging to link behavioral function to such a diminutive region. Among the most relied upon approaches to inferring its function have been by assessing its connections, interactions, cell types, and shape to draw inferences which can be quantified.

For a combinatory connectomics analysis to succeed in the claustrum, changes will need to occur in the neuroimaging status quo. The claustrum literature is woefully lacking *in vivo* imaging, as well as human imaging. Regardless of species, sample sizes will need to increase dramatically, in order to help overcome the problem of individual differences that plagues neuroimaging data. Intra-individual differences should also be addressed, as there have been very few adjustments made to account for the growing evidence that connectivity is state-dependent. In small structures, tweaks in an imaging protocol can have a far more pronounced affect; therefore, it is vital that claustrum studies utilize the highest available resolution of MR images, and the highest number diffusion gradients. Including a claustrum label in any of the parcellation software atlases would drastically improve the reliability of the literature.

## Conclusions

After centuries of research and much recent interest, a comprehensive theory of the claustrum's role in the brain and its sensory sub-networks remains lacking. Yet, the field of macro-scale connectomics—made possible through the refinement of neuroimaging and computational methods—provides a new basis for exploring intricate patterns of neural wiring and has already enabled broad-scale exploration of many major brain structures and pathways. The advancing sophistication of brain imaging and computational approaches is ideally suited for understanding the relative connectomics contributions of smaller brain regions which have only begun to be examined via brain mapping methods. Even the seemingly simple mathematical question of whether the claustrum is more crucial in inter- or intra- network connectivity remains unanswered and worthy of examination. Understanding whether the claustrum participates in each sub-network to an equal quantitative extent would allow assessment of whether the structure performs a single function for each network or multiple functions, unique to each network. Comparison of claustral network metrics throughout human development could illuminate considerable information about the establishment and re-enforcement of inter- and intra-hemispheric communication. Finally, macro-connectomics analysis of a region of such growing popularity could set a leading precedent for investigating small structures and sub-structures of the brain.

In conclusion, the purpose of the claustrum—of interest to brain science for centuries—stands a chance of being unlocked through the use of macro-scale connectomics methods. While the study of neuronal connections is far from novel, the increasingly complex statistical algorithms for understanding the functions of network-wide interactions remain in their infancy. Furthermore, studies which combine microscopic detail with macro-scale investigation of multiple brain regions remain rare at present, if not unheard of. Such a wide scope of analysis would likely enhance the knowledge of almost any brain region; however, the putatively abstract and as yet unconfirmed role of the claustrum, beyond when considered in isolation, poses a valuable opportunity for connectomic analysis to break new ground and demonstrate its function by evaluating this stubbornly enigmatic structure across all scales. Likewise, multi-scale analysis of this under-appreciated brain region could demonstrate how much more robust our understanding becomes when networks and sub-networks are considered at all levels, rather than assuming that we can achieve this understanding through an additive process of examining individual micro-scale architecture. It is true that connectomic analysis is already enhancing our understanding of the brain. Yet, the advantages of these computational approaches seem underwhelming when merely used to recapitulate understanding of established networks. It remains unlikely—with contemporary technologies alone—that this mysterious strip of non-cortical, non-subcortical matter nestled deep within the cerebrum will ever be understood without applying connectomic network theory at the broadest possible scales.

### Conflict of interest statement

The Associate Editor Mihail Bota declares that, despite being affiliated to the same institution as authors, the review process was handled objectively and no conflict of interest exists. The authors declare that the research was conducted in the absence of any commercial or financial relationships that could be construed as a potential conflict of interest.
